# A 12-year epidemiological study of *Acinetobacter baumannii* from blood culture isolates in a single tertiary-care hospital using polymerase chain reaction (PCR)–based open reading frame typing

**DOI:** 10.1017/ash.2022.279

**Published:** 2022-08-08

**Authors:** Yuji Fujikura, Takaaki Hamamoto, Atsushi Yuki, Ayumi Sampei, Nozomi Ichie, Kazuho Takamizawa, Sakika Nomura, Yusuke Serizawa, Tomohiro Ohno, Hironori Tsujimoto

**Affiliations:** 1 Department of Medical Risk Management and Infection Control, National Defense Medical College Hospital, Tokorozawa, Saitama, Japan; 2 Division of Infectious Diseases and Respiratory Medicine, Department of Internal Medicine, National Defence Medical College, Tokorozawa, Saitama, Japan; 3 Department of Clinical Laboratory, National Defence Medical College Hospital, Tokorozawa, Saitama, Japan

## Abstract

**Objective:**

*Acinetobacter baumannii* is a causative agent of healthcare-associated infections, and the introduction and spread of *A. baumannii* that has acquired drug resistance within a hospital are serious healthcare problems. We investigated the transition of epidemic clones and the occurrence of outbreaks by molecular epidemiological analysis to understand the long-term behavior of *A. baumannii* within a single facility.

**Methods:**

*A. baumannii* isolates collected from blood-culture–positive patients between January 2009 and December 2020 were subjected to PCR-based open reading frame typing (POT) for species identification, clonal typing, and homology searches.

**Results:**

Of the strains isolated from blood cultures, 49 were identified as *A. baumannii* and analyzed with POT. The POT#1=122 clones had different antimicrobial resistance profiles to the other POT clones, and strains belonging to this clone were dominant during outbreaks of multidrug-resistant *Acinetobacter*. Although the clonal diversity of *A. baumannii* decreased and its antimicrobial resistance increased during the outbreaks, clonal diversity and the in-hospital antibiogram improved at the end of the outbreaks. The POT#1=122 clone was not eliminated from the hospital during the study period.

**Conclusions:**

POT is a simple and suitable method for molecular epidemiological monitoring and can show the introduction, outbreak, and subsequent transition of an epidemic clone of *A. baumannii.*

*Acinetobacter baumannii*, an aerobic gram-negative rod bacterium, is a ubiquitous pathogen in healthcare-associated nosocomial infections, and the progression of antimicrobial resistance is particularly concerning.^1–3^ The introduction and spread of certain *Acinetobacter* clones that have acquired drug resistance within a facility^4,5^ can alter the in-hospital antibiogram, leading to the development of carbapenem resistance^6^ and causing occasional outbreaks of multidrug-resistant *Acinetobacter* (MDRA) strains.

In some countries, MDRA is detected at a high frequency^7^; in Japan in 2020, its prevalence was low, with an in-hospital MDRA detection rate of 92 (0.35%) of 25,980 isolates.^8^ In countries where MDRA is infrequent, few reports are available on the long-term transition of epidemic clones and antibiograms in facilities in which drug-resistant *A. baumannii* has been introduced or outbreaks have occurred.

Molecular epidemiological analysis is important for monitoring such outbreaks,^9^ but it requires time-consuming methods such as multilocus sequence typing (MLST) and pulsed-field gel electrophoresis (PFGE).^10^ These methods take several days to perform and require specialized software and extensive experience to analyze and interpret band patterns. Conversely, PCR-based genotyping methods or PCR-based open reading frame typing (POT) methods,^11^ which utilize the detection of open reading frame diversity specific to a bacterial genetic lineage or species, have been developed using multiplex PCR approaches. The suitability of POT for identifying clones and evaluating strain homology has been reported.^12–14^ POT kits are now commercially available and facilitate molecular epidemiological surveillance in general laboratories.

Our hospital experienced MDRA outbreaks in 2012–2014 and 2016–2018. In this study, we investigated the evolution of the epidemic clones and changes in the institutional antibiogram throughout these MDRA outbreaks by molecular epidemiological analysis using blood-culture isolates to determine the long-term behavior of *A. baumannii* within a single hospital and the usefulness of POT to monitor such outbreaks.

## Methods

### Bacterial isolate collection

This study was conducted at the National Defense Medical College, an 800-bed tertiary-care hospital in Japan. Consecutive *A. baumannii* clinical isolates were collected from patients with at least 1 positive blood culture detected by the hospital laboratory between January 2009 and December 2020. For bacterial identification and to assess antimicrobial susceptibility, standard blood-culture systems were used. BACTEC9240 (Beckman Coulter, Tokyo, Japan) was used until February 2019, and BACTEC-FX (Beckman Coulter) was used after March 2019. Microscan Walkaway 96 Plus (Beckman Coulter) with Neg Combo Panel (Neg Combo 6.1J) was used until March 2015, and Microscan Walkaway 96 Plus (Beckman Coulter) with Neg NF Combo 1J was used from April 2015 to March 2019. Microscan Walkaway 96 Plus (Beckman Coulter) with Neg Combo NF 3J was used from April 2019 onward. This study conformed to the principles of the Declaration of Helsinki and was approved by the institutional review board (approval no. 4502).

### Antimicrobial susceptibility

Susceptibility was interpreted according to the most recent guidelines of the Clinical and Laboratory Standards Institute. Isolates with intermediate susceptibility were considered resistant. In accordance with the Japanese Ministry of Health, Labour, and Welfare, multidrug resistance was defined as resistance to the following three classes of antibiotics: carbapenems (imipenem-cilastatin or meropenem), fluoroquinolones (ciprofloxacin or levofloxacin), and amikacin.

### POT method

All samples were analyzed using a commercially available *Acinetobacter* POT kit (Cica Geneus Acineto-POT-Kit, Kanto Chemical, Tokyo, Japan). Isolates were cultured for 24 hours on sheep-blood agar plates, and genomic DNA was extracted with a Cica Geneus DNA Extraction Kit (Kanto Chemical). Multiplex PCR assays were performed according to the manufacturer’s instructions using a mixture of 8 μL template DNA, 4 μL Apta Taq DNA Master, 4 μL PCR supplement, and 4 μL primer mixture. Two primer mixtures were used because 2 multiplex PCR reactions were required. The primer target regions were composed of *atpA*, OXA-51, 7 genomic islets and 12 genomic islands, and a specific gene other than an *A. baumannii* gene for species identification.

Multiplex PCR reactions were started with initial denaturation at 94°C for 15 seconds and then subjected to 30 cycles of 94°C for 15 seconds and 60°C for 180 seconds. The PCR products were electrophoresed on 4% agarose-gels in 0.5× tris-borate EDTA at 100 V for 45 minutes. The bands were then visualized with ethidium bromide.

Three POT numbers were provided for the band pattern of each sample (POT#1-POT#2-POT#3). Depending on the presence or absence of the amplified DNA band, they were scored in the order of PCR amplicon sizes as either “+” = “1” or “−” = “0” (binary code). Each binary band code was multiplied by 2^n^ (n = 6 to 0), and the total was calculated. For example, the binary code of sequence type 2 (1111010) was converted to 122 as follows: (1 × 64) + (1 × 32) + (1 × 16) + (1 × 8) + (0 × 4) + (1 × 2) + (0 × 1).^11^ These steps were repeated for the other multiplex PCR procedures, and the series of POT numbers was converted according to the manufacturer’s instructions (Appendix Fig. 1).

### Outbreak periods

Because MDRA is infrequent in Japan, the beginning of an outbreak was defined as the occurrence of 2 or more MDRA-positive patients in 1 month. The end of an outbreak was defined as 3 consecutive months without MDRA detection. According to these definitions, the first outbreak observed at our hospital was from August 2012 to March 2014 and the second was from July 2016 to May 2018.

### Statistical analysis

The χ^2^ test was used for noncontinuous variables and the Fisher exact test was used for small sample sizes. All statistical analyses were performed using JMP Pro version 14.2 software (SAS Institute, Cary, NC).

## Results

Between January 2009 and December 2020, 89 cases were identified as positive for *A. baumannii* by routine laboratory tests from blood cultures. POT analysis was performed on these samples, except for *A. pittii*, *A. nosocomialis*, and other species. In total, 49 cases (55.1%) were identified as positive for *A. baumannii* and the POT type was determined.

**Fig. 1.**
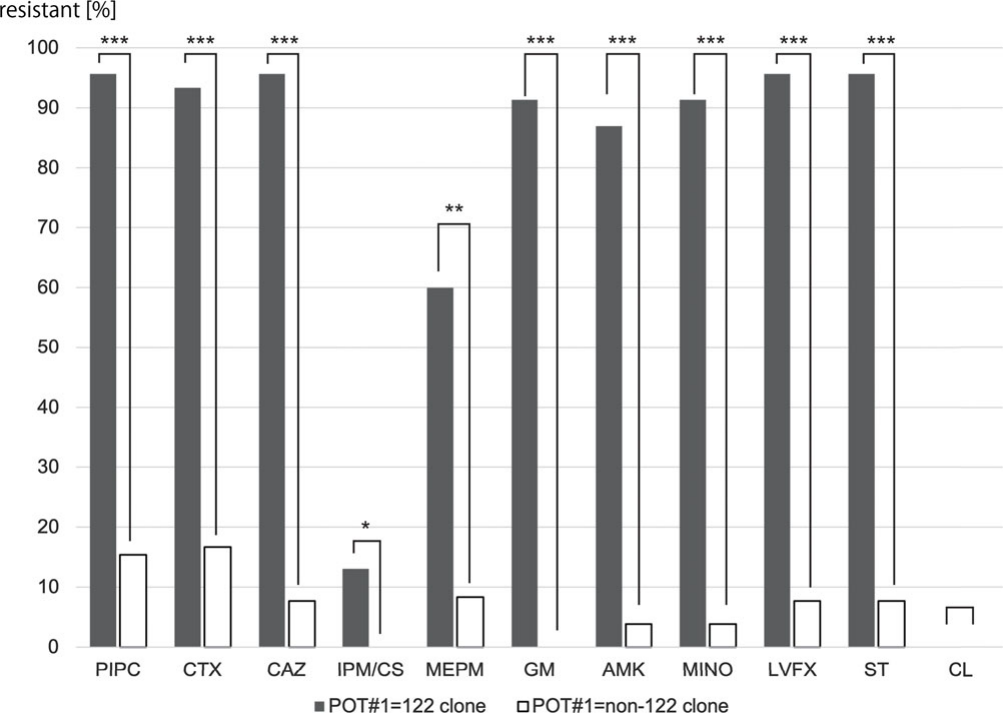
Antimicrobial susceptibility between the POT#1=122 clone and POT#1=non-122 clones. *Note.* *P < .05; **P < .01; ***P < .001. AMK, amikacin; CAZ, ceftazidime; CL, colistin; CTX, cefotaxime; GM, gentamicin; IPM/CS, imipenem/cilastatin; LVFX, levofloxacin; MEPM, meropenem; MINO, minomycin; PIPC, piperacillin; POT, PCR-based open reading frame typing; ST, sulfamethoxazole-trimethoprim.

The detection date, POT type (#1-#2-#3), and antimicrobial resistance of each sample are listed in Table 1. The drug-resistance profile of the POT#1=122 clone was different from that of the other clones (Fig. 1). All POT#1=122 clones except colistin showed a trend toward significant drug resistance. In our hospital, MDRA strains that were isolated from sputum and urine during the outbreak belonged to either 122-26-55, 122-24-55, or 122-16-54.^12^ These POT strains were also detected in the blood-culture specimens analyzed in this study. Some strains of the same type involved in the outbreak were susceptible to carbapenems, and others were initially resistant to levofloxacin and amikacin, but they later converted to MDRA.

**Table 1.** POT and Antimicrobial Susceptibility

Sampling Date	POT#1	POT#2	POT#3	Drug Resistance										
				PIPC	CTX	CAZ	IPM/CS	MEPM	GM	AMK	MINO	LVFX	ST	CL
Jul 2009	104	13	0	S	S	S	S	…	S	S	S	S	S	…
Aug 2009	44	41	34	S	S	S	S	S	S	S	S	S	S	…
Feb 2010	64	1	10	S	S	S	S	…	S	S	S	S	S	…
Feb 2010	8	13	0	S	S	S	S	…	S	S	S	S	S	…
Jul 2010	8	13	0	S	S	S	S	…	S	S	S	S	S	…
Oct 2010	8	13	0	S	S	S	S	…	S	S	S	S	S	…
Nov 2010	8	13	0	S	S	S	S	…	S	S	S	S	S	…
Nov 2010	105	9	0	R	R	S	S	…	S	S	S	S	S	…
Feb 2011	8	13	2	S	S	S	S	…	S	S	S	S	S	…
Mar 2011	72	1	8	S	S	S	S	…	S	S	S	S	S	…
Apr 2011	44	41	32	S	S	S	S	S	S	S	S	S	S	…
Jul 2011	122	26	55	R	R	R	S	S	R	R	R	R	R	…
Aug 2011	104	45	44	R	R	R	S	…	S	S	S	R	R	…
Aug 2011	122	41	0	S	S	S	S	S	S	S	S	S	S	…
Aug 2011	122	45	44	R	R	R	S	…	R	R	R	R	R	…
Oct 2011	8	12	0	R	S	S	S	S	S	S	S	S	S	…
Dec 2011	121	5	0	S	S	S	S	…	S	S	S	S	S	…
Jan 2012	122	26	55	R	R	R	S	…	R	R	R	R	R	…
Feb 2012	122	26	55	R	R	R	S	…	R	R	R	R	R	…
Aug 2012	122	24	55	R	R	R	S	…	R	R	R	R	R	…
Oct 2012	122	26	55	R	R	R	R	R	R	R	R	R	R	…
May 2013	122	26	55	R	R	R	R	R	R	R	R	R	R	…
Jul 2013	8	13	0	S	S	S	S	…	S	S	S	S	S	…
Sep 2013	122	24	54	R	R	R	S	…	R	R	R	R	R	…
Nov 2013	122	26	55	R	R	R	S	R	R	R	R	R	R	…
Jun 2014	8	4	0	R	R	R	S	R	R	R	R	R	R	…
Jul 2014	122	26	55	R	R	R	S	…	R	R	R	R	R	…
Jul 2014	8	13	0	S	S	S	S	…	S	S	S	S	S	…
Aug 2014	122	24	54	R	R	R	S	…	R	R	R	R	R	…
Sep 2014	57	9	0	S	S	S	S	…	S	S	S	S	S	…
Sep 2014	122	24	54	R	R	R	S	…	R	R	R	R	R	…
Nov 2014	122	24	54	R	R	R	S	R	R	R	R	R	R	…
Dec 2014	122	26	55	R	R	R	S	R	R	R	R	R	R	…
Apr 2015	8	13	0	S	…	S	S	S	S	S	S	S	S	S
Jun 2015	8	13	0	S	…	S	S	S	S	S	S	S	S	S
Oct 2015	75	13	0	S	…	S	S	S	S	S	S	S	S	S
Nov 2015	122	24	54	R	…	R	S	S	R	R	R	R	R	S
Jun 2016	0	12	0	S	…	S	S	S	S	S	S	S	S	S
Jul 2016	122	26	55	R	…	R	S	S	R	R	R	R	R	S
Jul 2016	122	26	55	R	…	R	S	S	R	R	R	R	R	S
Jul 2016	122	24	54	R	…	R	R	R	R	R	R	R	R	S
Aug 2016	122	26	55	R	…	R	S	R	R	R	R	R	R	S
Sep 2016	122	26	55	R	…	R	R	R	R	R	R	R	R	S
Jan 2017	93	40	32	R	…	S	S	S	S	S	S	S	S	…
Apr 2018	44	40	32	S	…	S	S	S	S	S	S	S	R	…
Feb 2019	8	12	0	S	…	S	S	S	S	S	S	S	S	…
Feb 2020	108	8	2	S	…	S	S	S	S	S	S	S	S	…
Aug 2020	122	8	38	R	…	R	S	R	R	S	R	R	S	…
Dec 2020	122	8	38	R	…	R	S	S	R	S	R	R	S	…

Note. AMK, amikacin; CAZ, ceftazidime; CL, colistin; CTX, cefotaxime; GM, gentamicin; IPM/CS, imipenem/cilastatin; LVFX, levofloxacin; MEPM, meropenem; MINO, minomycin; PIPC, piperacillin; POT, PCR-based open reading frame typing; R, resistant; S, sensitive; ST, sulfamethoxazole-trimethoprim. Samples shaded in gray were detected during the outbreak periods.

**Fig. 2.**
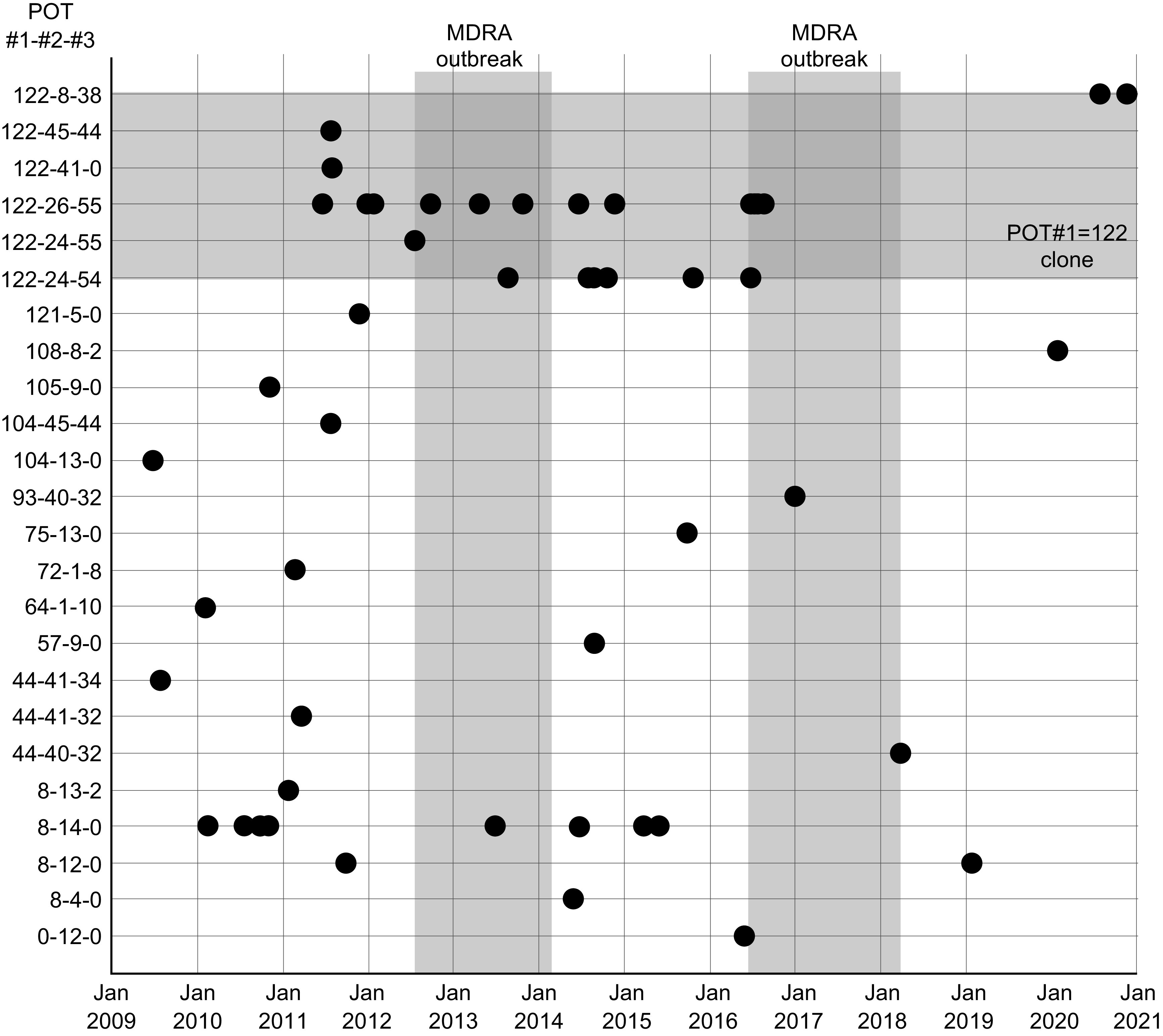
Detection date and POT diversity. Vertical gray bars indicate the outbreak periods, and the horizontal gray bar indicates the POT#1=122 clone. Note. POT, PCR-based open reading frame typing.

Figure 2 shows the dates when each strain was detected and the accumulation of POT#1=122 clones showing antimicrobial resistance. Before MDRA was detected, drug-resistant POT#1=122 clones had already been detected sporadically in our hospital, but during the outbreaks, POT#1=122 clones were predominant. The Shannon indices of the outbreak periods and the inter-outbreak period were 1.78 and 4.04, respectively. The Simpson diversity indices were 0.611 and 0.916 for the same periods, respectively, indicating lower clonal diversity during the outbreak periods.

**Fig. 3.**
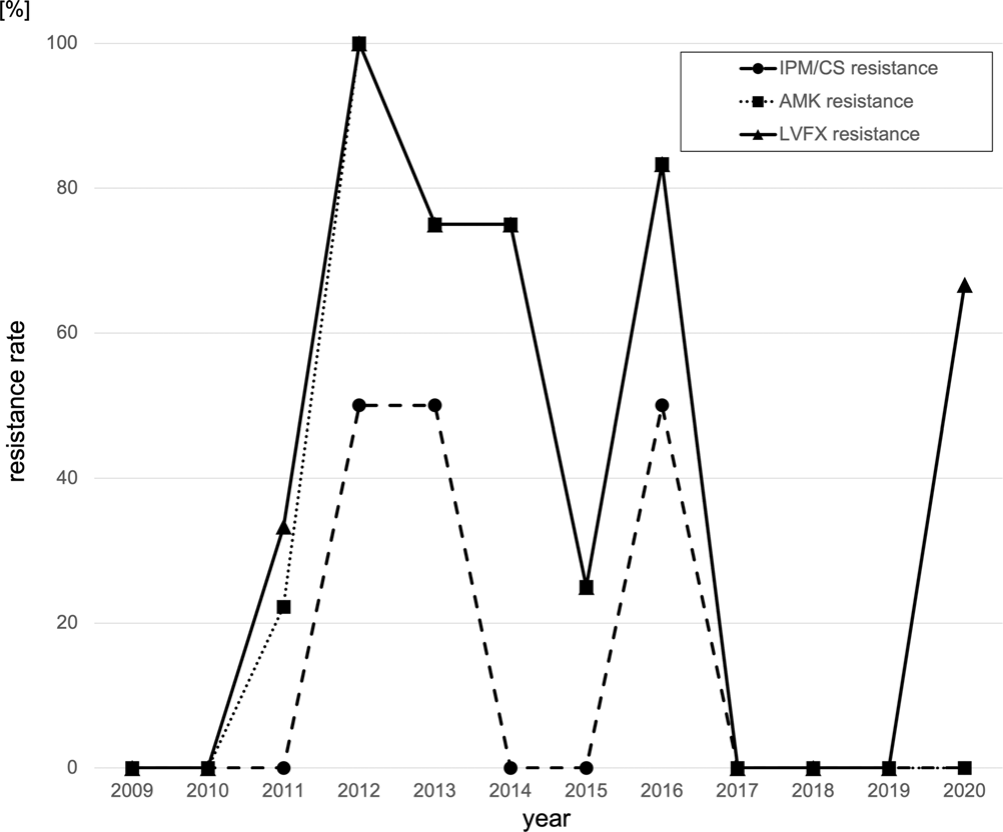
Chronological transition of the drug-resistance rate.

Finally, the antimicrobial resistance rates for imipenem-cilastatin, levofloxacin, and amikacin are shown by year in Figure 3. Drug-resistance rates increased significantly prior to both outbreak periods, but susceptibility showed a trend of improvement after the outbreaks. However, the POT#1=122 clone continues to be detected in our hospital and the resistance rate to amikacin has increased again since 2020.

## Discussion

In this study, we investigated the molecular epidemiological transition of *A. baumannii* at a single hospital over >10 years. We observed the loss of clonal diversity due to the introduction of drug-resistant epidemic clones into our hospital and the occurrence of outbreaks,^5^ as well as the recovery of clonal diversity after the outbreaks, along with changes in the in-hospital antibiogram. Interestingly, the epidemic clone was not eliminated and remained in the facility, suggesting that outbreaks may occur in the future and that continued surveillance is therefore warranted. Furthermore, POT was an effective surveillance method in terms of convenience and strain preservation.

The epidemiology of antimicrobial resistance of *Acinetobacter* differs among countries.^7^ Although the drug-resistance rate of *Acinetobacter* in Japan is low,^8^ MDRA outbreaks have been reported.^15^ Molecular epidemiology has shown that specific epidemic clones are prevalent within facilities during MDRA outbreaks,^4,5^ and the effect of continuously monitoring epidemic clones as part of a bundled approach to prevent outbreaks has also been noted.^2,16^ POT not only can determine a specific epidemic clone but also can evaluate strain homology in detail. POT can be used to observe the trends of specific strains and the diversity of clones in a hospital. The results of this study highlight the utility of the method.^12–14^

In molecular epidemiological analysis, MLST has been a common approach for determining epidemic clones. In addition, PFGE has been used to evaluate the association between strains detected during outbreaks.^10,17^ However, PFGE is less convenient than POT in that it requires simultaneous analysis of all potentially related strains, which is time-consuming and expensive. POT can be used for clone determination and to assign a strain-specific number by a simple multiplex PCR reaction, providing quantitative and objective unique data even if the strains are not preserved. The data can also be stored easily. POT is suitable for long-term monitoring because it allows easy comparisons of previous and new clinical specimens.^12^

The POT#1=122 clone that entered our facility had a distinctly different drug-resistance profile from the other clones identified and was the primary clone responsible for outbreaks at our hospital. POT#1=122 belongs to International Clone-2,^11,14^ which tends to become persistent in the hospital environment^18^ and to acquire drug resistance,^19^ which also makes it an outbreak-causing clone in an institution. At the time of both outbreaks, our hospital took a bundled approach,^20^ including thorough cleaning of the environment. However, the persistent detection of this clone means that it has not been eliminated. It is presumed to have persistently colonized the environment and to be carried in a certain number of patients attending our hospital.^5,19^

In-hospital antibiograms of *A. baumannii* showed that its resistance rate increased prior to the outbreaks. Furthermore, there was a temporary deterioration in resistance during these outbreaks, which improved afterward. When the frequency of drug-resistant epidemic clones is socially low, it is possible that the in-hospital antibiogram will improve when the outbreak subsides and the diversity of clones that remain susceptible will be restored. Conversely, in our hospital, the epidemic clones from 2019 that acquired antimicrobial resistance were detected more frequently and increased the rate of antimicrobial resistance, indicating the need for careful monitoring to prevent future outbreaks of MDRA.

This study had several limitations. First, epidemiological data are only partially reflected, especially because our analysis was limited to blood cultures. However, the use of only blood-culture strains did provide information on molecular epidemiological trends at our hospital. Second, because this was a retrospective study and there were limitations in the collection of specimens, the data are similarly partial. These limitations could be resolved in the future by performing POT in all cases, regardless of specimen type, when *A. baumannii* is detected.

In this study, POT was able to show the introduction, outbreak, and subsequent transition of an epidemic clone of *A. baumannii*, which can readily acquire drug resistance and cause an outbreak within a single facility. POT analysis showed that during an outbreak, the overall diversity of *A. baumannii* in the facility disappeared and a specific epidemic clone emerged, and that the diversity of the clones recovered again after a certain period. However, specific epidemic clones are not eliminated from a hospital, and it is highly likely that clones with characteristics that make them more likely to spread in an institution cannot be eliminated. POT is a simple and useful method to monitor the presence of such clones in an institution prior to and during an MDRA outbreak.

## Supplementary material

For supplementary material accompanying this paper visit https://doi.org/10.1017/ash.2022.279. click here to view supplementary material
