# A 12-year epidemiological study of *Acinetobacter baumannii* from blood culture isolates in a single tertiary-care hospital using polymerase chain reaction (PCR)–based open reading frame typing – ERRATUM

**DOI:** 10.1017/ash.2022.295

**Published:** 2022-09-02

**Authors:** Yuji Fujikura, Takaaki Hamamoto, Atsushi Yuki, Ayumi Sampei, Nozomi Ichie, Kazuho Takamizawa, Sakika Nomura, Yusuke Serizawa, Tomohiro Ohno, Hironori Tsujimoto

In the above article, the title has been edited to read “A 12-year epidemiological study of *Acinetobacter baumannii* from blood culture isolates in a single tertiary-care hospital using polymerase chain reaction (PCR)–based open reading frame typing”, correcting the spelling error of the word “polymerase”; the publisher apologizes for this error.
