# Supplementary material for: A 12-year epidemiological study of Acinetobacter baumannii from blood culture isolates in a single tertiary-care hospital using polymerase chain reaction (PCR)–based open reading frame typing
Source: Antimicrob Steward Healthc Epidemiol. 2022 Aug 8;2(1):e136. doi: 10.1017/ash.2022.279 (PMC9726563; doi:10.1017/ash.2022.279)
Supplement: Supplementary file 1 [file ashsup.zip › S2732494X22002790sup002.docx]

Appendix Figure 1. PCR-based open-reading frames typing (POT) method

In the POT method, the presence or absence of bands differentiates the species related to *Acinetobacter baumannii*: in Reaction 1, the product of 465 bp corresponds to A. baumannii, 401 bp to *A. pittii*, 362 bp to *A. nosocomialis*, 321 bp corresponds to A. sp. close to 13TU.

And two multiplex PCR reactions provide a series of POT numbers converted as illustrated. In reaction 1, the presence or absence of seven PCR products in the range of 81 to 271 bp is represented by a binary number of 1 and 0, arranged in descending order, and converted into decimal notation. This is applied to the range of 81 to 457 bp of POT 2 and POT 3 components of reaction 2 on the right in the same way, and the POT numbers are defined by a combination of three numbers POT 1, 2, and 3.
