# Supplementary material for: A 12-year epidemiological study of Acinetobacter baumannii from blood culture isolates in a single tertiary-care hospital using polymerase chain reaction (PCR)–based open reading frame typing
Source: Antimicrob Steward Healthc Epidemiol. 2022 Aug 8;2(1):e136. doi: 10.1017/ash.2022.279 (PMC9726563; doi:10.1017/ash.2022.279)
Supplement: Supplementary file 1 [file ashsup.zip › S2732494X22002790sup001.pdf]

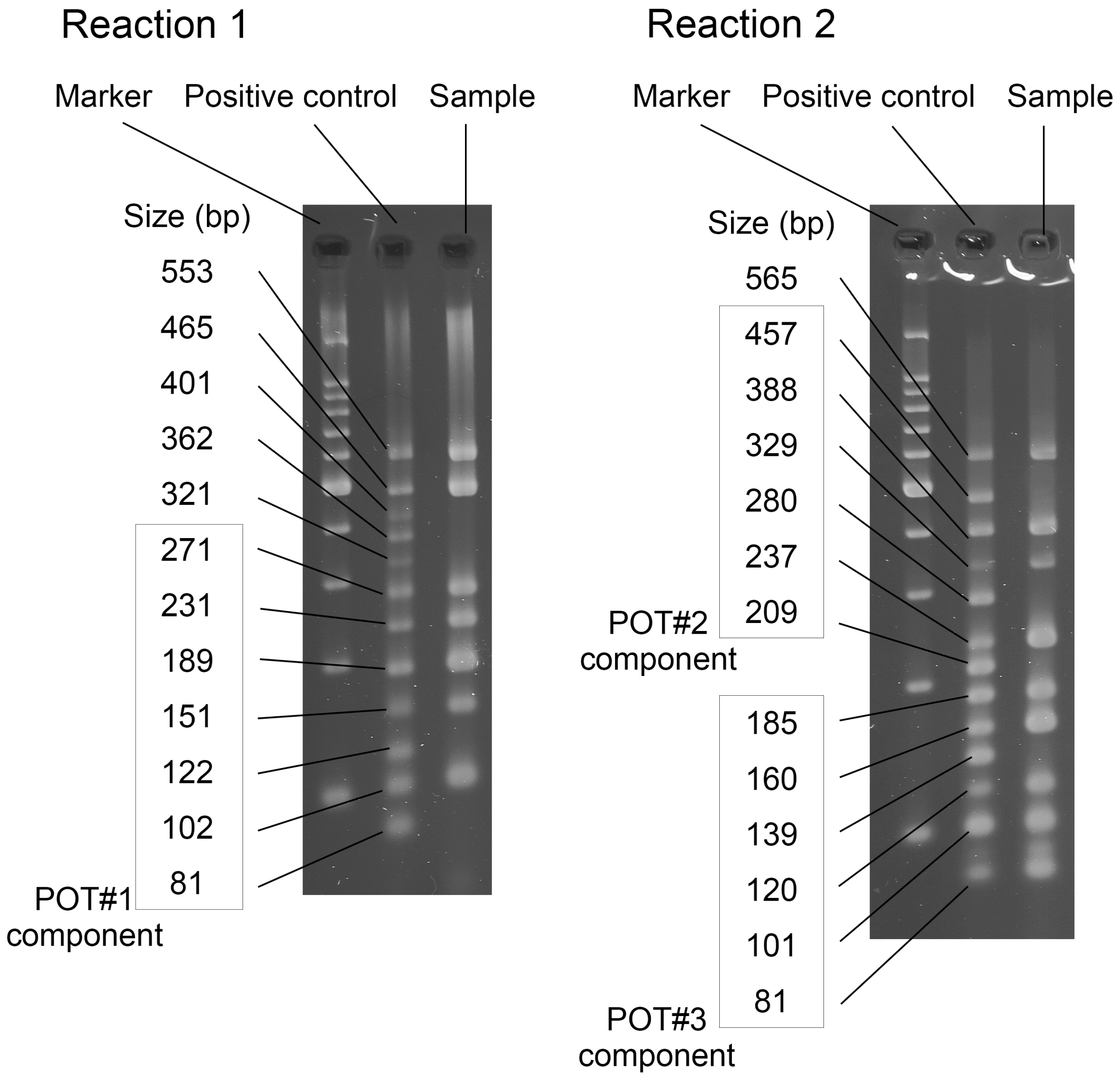

POT number (POT#1 - POT#2 - POT#3): “122 - 26 - 55”

POT#1: $1 \times 64 + 1 \times 32 + 1 \times 16 + 1 \times 8 + 0 \times 4 + 1 \times 2 + 0 \times 1 = 122$

POT#2: $0 \times 32 + 1 \times 16 + 1 \times 8 + 0 \times 4 + 1 \times 2 + 0 \times 1 = 26$

POT#3: $1 \times 32 + 1 \times 16 + 0 \times 8 + 1 \times 4 + 1 \times 2 + 1 \times 1 = 55$
